# Cleaning the Teeth

**Published:** 1869-09

**Authors:** J. P. Holmes


DENTAL REGISTER.

VOL. XXIII.] SEPTEMBER, 1869. [NO. 9.

### Original Communications.

### CLEANING THE TEETH.

BY J. P. HOLMES, D.D.S.

THIS subject has been but slightly noticed by the journals and by many Dentists. It may seem of little importance, yet, is very essential in many respects. The first point to be noticed is the cleanliness of the mouth which is often tainted very strongly from unclean teeth. Second, when unclean, the appearance of the teeth is much marred, which detracts from the appearance of the person. Third, when the teeth are not cleansed, tartar will accumulate, covering the teeth in some instances, all over quite thick, causing absorption of the gums, alveolar process, and finally the loss of the teeth. It is evident, then, that the teeth are lost from not being cleansed, thus showing the importance of this operation.

Thousands of teeth are yearly sacrificed, for the simple reason that they are not cleansed. It is therefore the duty, and to the interest of every honest and good Dentist to endeavor to save as many teeth as possible. In order to accomplish this object, care should be taken to fill the teeth as perfectly as possible, then finish completely, giving the "grand finale" by thoroughly cleaning the teeth of all foreign substances, stains, &c. When this is done, and instructions given to the patient to keep them in this condition, then is the operation complete, and not without.

It is an established fact that the teeth decay about fillings, and the latter come out from neglect of the brush. That would not be so if the proper attention had been given, showing to the Dentist that it is duty and to his interest to have the teeth of his patients kept clean, in order that his work may be permanent and remain in the mouth.

The work done in a mouth is shown to a greater advantage, and the patient is more than pleased at the metamorphosis, and exclaims, how nicely my mouth feels, and how white you have made my teeth. This operation is always appreciated by the patient, and I have noticed the pleasant surprise depicted on the patients face when, on handing them the mirror, they perceive the sudden change from dark or black stained teeth to white, clean teeth, and the effect produced in the general appearance of the person.

It is no easy or very nice operation to clean a set of teeth thoroughly, it requires time, energy, patience and hard work to do justice to the patient. My mode of cleaning the teeth is as follows:

1st. Remove all tartar by use of scalers, using, if there is much tartar, and breath bad, phenol sodique, diluted two-thirds water, throwing it on gums and teeth with syringe used for this purpose. This disinfects the breath, making it more pleasant for the patient, and operator, also, checks the bleeding produced by scalers coming in contact with the gums, in search of tartar. After removing all the tartar, next proceed to clean the stain from the teeth, by use of very fine pumice stone, used on very soft white pine wood, made in different shapes, etc. Then select a tooth for a model and clean it as as nicely as possible. Then proceed to next, etc., until all are clean like model. After this, go over all the teeth with tooth powder and soap, giving them a genteel brushing with the patients brush. This will clean the teeth thoroughly, and make them look white and nice. Rinse the mouth out and apply on a pledget of cotton, to the gums where tartar has been, a solution of creasote and iodine, equal parts, diluted with alcohol one half. This will make gums heal nicely. Where there is a good deal of tartar, gums diseased bleed from slight touch. It requires three to five sittings to remove all the tartar and cure the gums. In cases of this kind, remove all tartar possible first sitting, applying freely over all the gums the above solution, discharging patient giving an astringent mouth wash to be used three or four times daily for two days. Then to return to have balance of tartar removed, continuing thus until the mouth is well. By pursuing this course nearly any case can be cured in a short time. As soon as gums are entirely cured clean as above.

I have been very successful in this way, winning many warm friends, and much work, by cleansing the teeth and saving them from destruction, when they had been given up for lost. The patient is very apt to take special care of the teeth after this, and will have them examined frequently and if they require attention will have it given them. Teeth that are often given up by patients as being too far gone to be saved, on examination are often found covered all over with tartar, gums diseased, breath offensive, etc., but teeth pretty good. Perhaps a few decayed, one or two may be beyond the skill of the Dentist. After a close examination you say to your patient, all your teeth except one or two can be saved by a little skill and energy. Patient consents to have them treated and filled. After being informed of the value of natural over artificial teeth, then proceed to finish; first, cleansing the mouth of all foreign substances, so as to be able to operate on the teeth, in filling, etc., and to the patients surprise you have brought order out of chaos. They find that they have a good, nice, white set of natural teeth, which have, perhaps, cost but little more than a set of artificial teeth.

Thus, by cleansing the teeth, you have saved the patient the loss of their natural teeth, and at the same time feel that you have done a good deed. A good price should be charged, and patients who appreciate their teeth, and have any appreciaticn of the good done, will be willing to compensate handsomely for the work, and be grateful in addition.

